# Associations of perceived teacher–student relationship and friendship quality with adolescents' interest in physical education: a latent profile analysis

**DOI:** 10.3389/fspor.2025.1677083

**Published:** 2025-11-13

**Authors:** Xinyi Chen, Wenying Huang, Chang Hu

**Affiliations:** Physical Education College, Jiangxi Normal University, Nanchang, China

**Keywords:** interest in physical education, teacher–student relationship, adolescents, friendship quality, latent profile analysis

## Abstract

**Objective:**

This study employed a person-centered Latent Profile Analysis (LPA) to explore adolescents' perceived teacher–student and friendship relationships in the school environment and to examine their association with interest in physical education.

**Methods:**

A survey was conducted among 3,613 adolescents using the Teacher–Student Relationship Scale, the Friendship Quality Scale, and the Interest in Physical Education Scale. LPA was applied to identify relationship quality profiles, and multinomial logistic regression was used to examine gender differences and associations with interest in physical education.

**Results:**

Three profiles emerged from the LPA: the Low Relationship Quality profile (23%, *n* = 855), the Moderate Relationship Quality profile (60%, *n* = 2,156), and the High Relationship Quality profile (17%, *n* = 602). Adolescents with lower levels of physical education interest were significantly more likely to belong to the Low Relationship Quality profile compared with the Moderate Relationship Quality profile [OR = 0.01, 95% CI (0.01, 0.02)]. In contrast, those with higher physical education interest were significantly more likely to belong to the High Relationship Quality profile [OR = 4.29, 95% CI (3.44, 5.35)]. In addition, males had higher odds of being classified into the High Relationship Quality profile than females [OR = 1.93, 95% CI (1.59, 2.35)]. Significant differences were observed across profiles on all dimensions of teacher–student and friendship relationships (all *p* < 0.001).

**Conclusion:**

Adolescents exhibit heterogeneous experiences of teacher–student and friendship relationship quality, which were significantly associated with differences in interest in physical education. By applying a person-centered approach, the study extends prior research by showing that teacher–student and friendship contexts are linked to adolescents' interest in physical education, underscoring the importance of considering interest as a distinct outcome in relational research.

## 1 Introduction

Adolescence represents a pivotal stage of physical and psychological development, during which students face academic pressures, transitions in school environments, and major changes in interpersonal relationships. Bronfenbrenner's ecological systems theory highlights that adolescents' development is embedded within multiple microsystems, among which the classroom and daily interactions serve as immediate contexts shaping their learning motivation and social adjustment (1). Schools, therefore, function not only as institutions for knowledge transmission but also as key contexts for socialization and personality development (2, 3). Within this environment, the quality of adolescents' relationships with teachers and peers profoundly influences their adjustment and development. Physical education, as a core domain of schooling, plays a particularly important role because it connects closely to adolescents' physical health, social competence, and the cultivation of lifelong exercise habits.

As one of the most influential microsystems in adolescents' development, the school and classroom environment play a central role in shaping students' learning motivation and behavioral engagement (4–7). Within this setting, interest in physical education represents a key form of intrinsic motivation that enhances enjoyment, promotes active participation, and supports cognitive and emotional growth (8–10). According to Self-Determination Theory (11), such interest reflects a fundamental element of intrinsic motivation and provides the psychological basis for sustained involvement in physical activity. Conversely, insufficient interest in physical education has been recognized as a major barrier to students' engagement in classes and long-term exercise participation. From the perspective of motivational theories, proactive behavior is primarily driven by internal motivation (12–14). From the perspective of motivational theories, proactive behavior is largely driven by motivation; thus, insufficient interest may lead to reduced participation in physical activities (15–17), highlighting the need to foster students' interest in this domain. Importantly, beyond curriculum design, the interpersonal quality of the school context—particularly supportive teacher–student relationships and high levels of friendship quality—has emerged as a decisive factor for developing and sustaining adolescents' interest in physical education (18–21).

The quality of teacher–student relationships and friendship quality constitutes a fundamental part of adolescents' psychosocial development (22–27). Vygotsky's sociocultural theory emphasizes that learning is inherently social: supportive teacher–student interactions and peer collaborations create a “zone of proximal development,” offering affective support and cognitive scaffolding (28). Within physical education, these relational dynamics are particularly salient. Teacher guidance enhances students' sense of security and belonging, while supportive peer relations foster enjoyment and persistence. Empirical studies further show that high-quality teacher–student relationships increase engagement and strengthen interest in physical education, whereas neglect or conflict undermines motivation and fuels negative attitudes (28–33). From an ecological perspective, friendship quality provides adolescents with emotional support and a sense of belonging, which not only facilitates social adjustment but also fosters greater interest and sustained engagement in learning activities, including physical education (34–36). Although ample evidence supports the positive role of teacher–student relationships and friendship quality in academic domains, their mechanisms in promoting students' interest in physical education remain underexplored. Given the unique social and embodied features of physical education classrooms, they represent a valuable yet insufficiently studied context for examining how the quality of teacher–student relationships and friendship quality specifically shape adolescents' interest in physical education.

Although teacher–student relationships and friendship quality have been widely examined in educational research, most evidence comes from studies on academic achievement or general learning motivation, whereas their role in cultivating students' interest in physical education has received far less attention (37–41). Previous investigations based on questionnaire surveys have primarily employed variable-centered approaches. For example, some studies have explored the associations between teacher–student relationship quality and students' peer relations (42–45), the mediating role of academic achievement or positive attitudes toward school in linking teacher–student relationship quality with prosocial behavior (4, 46, 47), and the correlations between teacher–student relationships, student interactions, and social participation in Dutch primary and secondary schools (48). These findings have been valuable for identifying specific pathways and independent associations. However, variable-centered approaches are limited in capturing the heterogeneity of relational dynamics across different student groups.

In contrast, person-centered approaches such as LPA allow researchers to identify distinct subgroups of students based on their response patterns and examine how these relational profiles differentially relate to motivational outcomes (49, 50). Applying such an approach is particularly relevant for physical education, because although the importance of teacher–student relationships and friendship quality has been well established in academic contexts, their role in shaping students' learning interest in PE remains understudied (4, 51–52). Viewing this issue through the lens of “interest” rather than general “motivation” highlights a gap in the existing literature that the present study seeks to address. Physical education classrooms are characterized by intense social interaction, immediate performance feedback, and strong peer influence, all of which may create distinct relational profiles among students. Person-centered analyses such as LPA can provide a more nuanced understanding of how diverse combinations of teacher–student and peer relations shape adolescents' interest in physical education, offering insights that variable-centered approaches might overlook.

Against this background, the present study addresses a gap in the literature by focusing on adolescents' interest in physical education. Specifically, it has two objectives. First, it seeks to identify distinct subgroups of students based on teacher–student relationship quality and friendship quality patterns, using a person-centered approach. Second, it examines how these relational profiles are associated with differences in students' interest in physical education. By pursuing these objectives, the study contributes to clarifying how interpersonal relationships shape adolescents' subject-specific interest in physical education and provides actionable implications for schools: fostering supportive teacher–student interactions and cultivating positive peer environments may enhance students' interest in physical education and encourage the establishment of lifelong physical activity habits.

## 2 Materials and methods

### 2.1 Participants and procedure

Before the main investigation, a pilot survey was conducted with 150 students in Jiangxi Province to evaluate the questionnaire's clarity, readability, and feasibility. Students were asked to report whether any items were confusing, difficult to comprehend, or ambiguous, and whether the response process was smooth and manageable. Feedback demonstrated that the questionnaire was clear and the procedures workable; therefore, no substantial modifications were required before launching the main survey.

A stratified convenience sampling strategy was used. In each of 12 provinces (Guangdong, Hunan, Guizhou, Henan, Guangxi, Yunnan, Chongqing, Sichuan, Shandong, Hubei, Hebei, and Jiangxi), one junior high school and one senior high school were selected, yielding 24 schools. Schools were chosen primarily based on accessibility and feasibility of collaboration, which allowed for broad geographic coverage but does not ensure national representativeness. Thus, the results should be generalized with appropriate caution.

The survey was conducted between September 10 and October 25, 2023. Questionnaires were created and hosted on the WJX online platform (https://www.wjx.cn/). Teachers received detailed training on the study's objectives, ethical principles, confidentiality requirements, and standardized administration procedures before data collection. This training ensured teachers were prepared to guide students through the process without influencing responses. After training, survey links and QR codes were distributed to two junior high school physical education teachers and two senior high school physical education teachers in each province. Under their supervision, students completed the electronic questionnaires independently in school computer laboratories.

To ensure sample rigor, inclusion criteria were: (1) full-time enrollment in junior or senior high school; (2) no history of mental illness, as confirmed via parental report in a health declaration form completed during informed consent; (3) no prior participation in similar studies, to avoid bias from repeated exposure; and (4) voluntary assent from students together with written informed consent from parents or guardians. Given the involvement of minors, ethical approval was obtained from the Ethics Committee of Jiangxi Normal University (IRB-JXNU-PEC-2023129). Students and their guardians were informed about anonymity, confidentiality, and their right to withdraw at any point without penalty.

Of the 5,898 responses initially collected, 1,050 questionnaires were excluded because more than 10% of items were unanswered. To ensure attentiveness, one non-scored check item—“Please select one option for this question”—was embedded in the survey. Responses not following this instruction were considered invalid, leading to the exclusion of 423 questionnaires. Following prior research practices (53, 54), cases with values that deviated more than ±3 standard deviations from the mean on key continuous variables were considered outliers and therefore removed, resulting in the exclusion of 312 cases. In order to facilitate future longitudinal follow-up, 500 questionnaires from students in terminal grades (junior high year 3 and senior high year 3) were excluded. After all quality-control procedures were applied, the final valid sample comprised 3,613 participants. These steps, combined with the use of validated measurement instruments, contributed to the reliability and validity of the dataset.

### 2.2 Measures

#### 2.2.1 Teacher–student relationship scale

The study employed the Chinese adolescent version of the Student–Teacher Relationship Scale validated by Zhu Xiaolin (55). The scale was originally adapted into Chinese by Wang Yun and Wang Xiaohua (56) for elementary school students and later revised by Zhu Xiaolin to suit adolescents in secondary schools. In the present study, this adolescent-specific Chinese version was explicitly applied to assess students' perceived relationship with their physical education teachers, rather than with teachers in general.

The Teacher–Student Relationship Scale consists of 18 items across four dimensions: Intimacy, Conflict, Attachment, and Avoidance. Responses are given on a 5-point Likert scale (1 = strongly disagree to 5 = strongly agree). Items from the negatively valenced dimensions (Conflict and Avoidance) were reverse-coded prior to scale construction, while Intimacy and Attachment items retained their original scoring direction. After reverse-coding, higher scores indicate a more positive perceived teacher–student relationship with physical education teachers.

Internal consistency in this study was satisfactory: total Cronbach's *α* = 0.847; Intimacy Cronbach's *α* = 0.742, Conflict Cronbach's *α* = 0.810, Attachment Cronbach's *α* = 0.785, and Avoidance Cronbach's *α* = 0.758 (see Section 2.2 for interpretation thresholds).

#### 2.2.2 Friendship quality scale

The Chinese version of the Friendship Quality Scale was administered, originally developed by Parker and Asher (57) and subsequently adapted for use in Chinese adolescent populations (58). The Friendship Quality Scale comprises 18 items measuring six dimensions: companionship and recreation, validation and caring, intimate disclosure and communication, help and guidance, conflict resolution, and conflict and betrayal.

In the original Parker and Asher version, items situate responses within everyday peer interactions (e.g., “How often do you and your friend…?”). In the Chinese adaptation, items are phrased more generally about adolescents' perceptions of their best friend or close friends, without embedding explicit situational vignettes. This adaptation ensures cultural appropriateness while retaining the core constructs.

Items are rated on a 5-point Likert scale (1 = strongly disagree to 5 = strongly agree). For scoring purposes, items from negatively valenced dimensions—i.e., conflict resolution, and conflict and betrayal—were reverse-coded. Higher total and subscale scores represent higher levels of perceived friendship quality.

Internal consistency in this study was good: total Cronbach's *α* = 0.851; companionship and recreation Cronbach's *α* = 0.732, validation and caring Cronbach's *α* = 0.737, intimate disclosure and communication Cronbach's *α* = 0.764, help and guidance Cronbach's *α* = 0.707, conflict resolution Cronbach's *α* = 0.610, and conflict and betrayal Cronbach's *α* = 0.724 (see Section 2.2 for interpretation thresholds).

#### 2.2.3 Interest in physical education scale

The study employed the Chinese version of the Interest in Physical Education Scale, adapted by Wang Xiaozan for Chinese students (59). The instrument assesses students' interest in learning physical education. It consists of 27 items grouped into four dimensions: level of sports participation, positive interest in physical education learning, degree of autonomous learning, and negative interest in physical education learning.

Each item is rated on a 5-point Likert scale (1 = strongly disagree to 5 = strongly agree). To maintain a consistent scoring direction, items in the negatively valenced dimension (negative interest in physical education learning) were reverse-coded prior to computing subscale and total scores. Higher scores, therefore, indicate a stronger interest in physical education learning.

Internal consistency in the present study was acceptable (Cronbach's *α* = 0.828). Subscale reliability coefficients were: level of sports participation, Cronbach's *α* = 0.899; positive interest in physical education learning, Cronbach's *α* = 0.870; degree of autonomous learning, Cronbach's *α* = 0.815; and negative interest in physical education learning, Cronbach's *α* = 0.856 (see Section 2.2 for interpretation criteria).

### 2.2 Data analysis

Data were analyzed using SPSS 27.0 and Mplus 8.0. Descriptive statistics were calculated for all study variables. For continuous variables that met the normality assumption, results were expressed as mean ± standard deviation; for categorical variables, frequency and percentage were reported.

Group differences in categorical demographic characteristics (e.g., gender, household registration) were examined using chi-square (*χ*^2^) tests, with Cramer's V reported as the effect size measure. For continuous variables, one-way analysis of variance (ANOVA) was used, with *η*^2^ coefficients reported as effect sizes.

LPA was conducted in Mplus 8.0 to identify unobserved subgroups of adolescents. LPA is a person-centered approach that identifies latent categorical subgroups based on participants' response patterns across multiple continuous indicators (49, 50). The observed indicators entered into the models included four dimensions of teacher–student relationship (intimacy, conflict, attachment, and avoidance) and six dimensions of friendship quality (companionship and recreation, validation and caring, intimate disclosure and communication, help and guidance, conflict resolution, and conflict and betrayal).

Model comparisons were conducted for one- to five-class solutions. Six fit indices guided model evaluation: Akaike Information Criterion (AIC), Bayesian Information Criterion (BIC), adjusted Bayesian Information Criterion (aBIC), Lo–Mendell–Rubin adjusted likelihood ratio test (LMRT), bootstrapped likelihood ratio test (BLRT), and entropy (60, 61). Model selection considered lower AIC, BIC, and aBIC values, significant LMRT and BLRT values, higher entropy values, parsimony, and theoretical interpretability.

After identifying the optimal latent profiles, ANOVA and *post hoc* tests were used to examine differences in interest in physical education among adolescents across latent profiles. ANOVA tested global differences, while *post hoc* tests specified pairwise contrasts.

Finally, in evaluating internal consistency, Cronbach's *α* coefficients were interpreted according to widely accepted thresholds: ≥0.70 acceptable, ≥0.80 good, ≥0.90 excellent (62, 63). These criteria were applied to both total scales and subscales.

## 3 Results

### 3.1 Common method bias

Since the focal variables of this study—teacher–student relationship, friendship quality, and interest in physical education—were all measured using self-report questionnaires, both procedural and statistical remedies were implemented to reduce potential common method bias.

For statistical verification, Harman's single-factor test was performed on the dataset. The unrotated exploratory factor analysis revealed 14 factors with eigenvalues greater than 1, and the largest single factor explained 16.30% of the variance. Because this value was well below the commonly adopted benchmark of 40%, the results indicated that a single factor did not account for most covariance among the measures (64, 65).

To further test common method variance, we conducted a confirmatory factor analysis (CFA) in which all observed variables were constrained to load on a single latent factor. The model fit was poor (*χ*^2^/df = 22.001, CFI = 0.386, TLI = 0.365, RMSEA = 0.074), suggesting no single factor dominated the covariance structure.

These results support the conclusion that common method bias was not a serious concern in the present study.

### 3.2 Model Fit evaluation and selection of latent profiles

The ten dimensions of the Teacher–Student Relationship Scale and the Friendship Quality Scale were used as observed variables. Potential profile models with 1–5 classes were extracted, and the fit indices for each model are shown in Table 1. Latent profile models with one to five classes were estimated, and the corresponding fit indices are reported in Table 1. The information criteria (AIC, BIC, aBIC) consistently decreased as the number of classes increased. The Lo–Mendell–Rubin (LMR) and bootstrap likelihood ratio (BLRT) tests were significant for all models, suggesting that each additional class improved model fit. However, the four- and five-class models produced entropy values below 0.80 and included very small or theoretically ambiguous groups, limiting their interpretability. The two-class model, though statistically acceptable, yielded overly simplistic profiles and failed to capture nuanced differences in relationship quality. By contrast, the three-class model demonstrated acceptable fit indices, retained an entropy value above 0.80, and showed a relatively balanced distribution of individuals across classes. Importantly, the three profiles could be meaningfully distinguished (i.e., high, moderate, and low quality) and aligned with previous findings on adolescent relationships, thereby offering both theoretical interpretability and practical applicability. On this basis, the three-class model was selected for subsequent analyses.

**Table 1:** Fit indices for latent profile models of teacher–student relationship and friendship quality.

Clusters	AIC	BIC	aBIC	Entropy	LMRp	BLRTp	Category probabilities
1-profile	109,169.243	109,293.089	109,229.539	/	/	/	
2-profile	104,268.473	104,460.434	104,361.931	0.846	<0.001	<0.001	0.25/0.75
3-profile	102,577.438	102,837.514	102,704.059	0.840	<0.001	<0.001	0.23/0.17/0.60
4-profile	101,996.906	102,325.098	102,156.690	0.757	<0.001	<0.001	0.19/0.30/0.37/0.14
5-profile	101,506.728	101,903.035	101,699.675	0.775	<0.01	<0.001	0.07/0.18/0.11/0.35/0.29

### 3.3 Three latent profiles of teacher–student relationship and friendship quality

**Figure 1:**
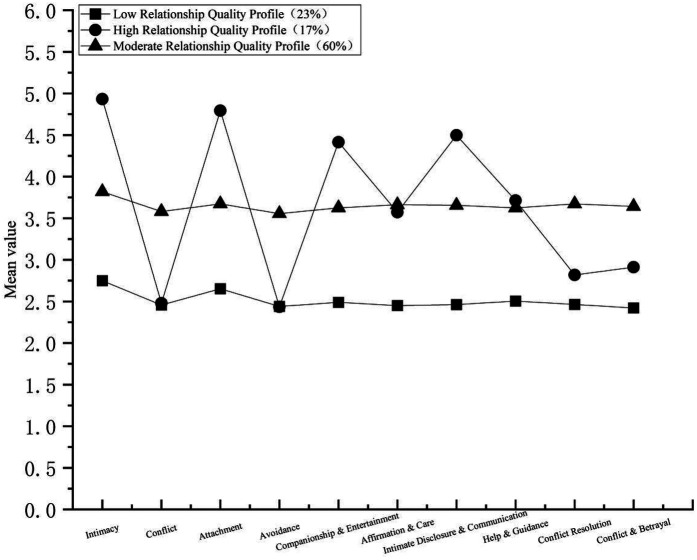
The latent profile model of teacher–student relationship and friendship quality.

Figure 1 illustrates the three identified profiles, displaying the mean levels across the four dimensions of Teacher–Student Relationship (intimacy, conflict, attachment, and avoidance) and the six dimensions of Friendship Quality (companionship & recreation, validation & caring, intimate disclosure & communication, help & guidance, conflict resolution, and conflict & betrayal).

The first profile, labeled the low relationship quality profile (*n* = 855, 23% of the sample), was characterized by the lowest scores on both Teacher–Student Relationship and Friendship Quality. The second profile, the high relationship quality profile (*n* = 602, 17%), achieved the highest scores on both measures. The third profile, labeled the moderate relationship quality profile (*n* = 2,156, 60%), showed intermediate scores between the low and high groups.

### 3.4 Distribution characteristics of LPA

Three distinct profiles were identified based on the mean scores for Teacher–Student Relationship and Friendship Quality dimensions (see Table 2). Adolescents in the first profile reported the lowest mean values across most dimensions (e.g., intimacy, attachment, companionship, affirmation, communication, and guidance) and the lowest scores in conflict resolution, conflict, and betrayal. Because the latter dimensions were reverse-coded, lower scores indicate poorer relationship quality. Therefore, this group (*n* = 855, 23%) was conceptualized as the Low-relationship quality profile.

**Table 2:** Comparison of teacher–student relationship and friendship quality dimensions across latent profiles.

Variable	Low relationship quality profile (*n* = 855)	High relationship quality profile (*n* = 602)	Moderate relationship quality profile (*n* = 2,156)	Test statistic	*P*	Effect size	Scheffe
Intimacy, M (SD)	2.62	4.01	3.69	*F*(2, 3,610) = 669.76	<0.001***	*η*^2^ = 0.225	2 > 3 > 1
	0.90	0.79	0.80				
Conflict, M (SD)	2.54	3.41	3.71	*F*(2, 3,610) = 554.26	<0.001***	*η*^2^ = 0.295	3 > 2 > 1
	0.85	1.15	0.79				
Attachment, M (SD)	2.53	3.87	3.54	*F*(2, 3,610) = 573.73	<0.001***	*η*^2^ = 0.248	2 > 3 > 1
	0.80	0.94	0.84				
Avoidance, M (SD)	2.51	3.37	3.69	*F*(2, 3,610) = 536.75	<0.001***	*η*^2^ = 0.257	3 > 2 > 1
	0.85	1.14	0.82				
Companionship & Entertainment, M (SD)	2.41	3.78	3.59	*F*(2, 3,610) = 574.1	<0.001***	*η*^2^ = 0.222	2 > 3 > 1
	0.88	1.02	0.92				
Affirmation & Care, M (SD)	2.43	3.55	3.66	*F*(2, 3,610) = 541.43	<0.001***	*η*^2^ = 0.235	3 > 2 > 1
	0.89	1.10	0.91				
Intimate Disclosure & Communication, M (SD)	2.37	3.88	3.62	*F*(2, 3,610) = 758.24	<0.001***	*η*^2^ = 0.261	2 > 3 > 1
	0.77	0.95	0.88				
Help & Guidance, M (SD)	2.49	3.73	3.62	*F*(2, 3,610) = 501.62	<0.001***	*η*^2^ = 0.218	2 > 3 > 1
	0.90	1.05	0.90				
Conflict Resolution, M (SD)	2.50	3.45	3.71	*F*(2, 3,610) = 450.83	<0.001***	*η*^2^ = 0.216	3 > 2 > 1
	0.99	1.18	0.95				
Conflict & Betrayal, M (SD)	2.45	3.55	3.68	*F*(2, 3,610) = 542.56	<0.001***	*η*^2^ = 0.246	3 > 2 > 1
	0.92	1.14	0.87				

1, Low relationship quality profile; 2, High relationship quality profile; 3, Moderate relationship quality profile; M, means; SD, standard deviation.

*** *p* < 0.001.

In contrast, adolescents in the second profile demonstrated the highest average scores on intimacy, attachment, companionship, intimate disclosure, and help and guidance, along with relatively high scores in the remaining dimensions. Their overall relationship indicators revealed consistently stronger quality compared to the other profiles. Accordingly, this profile (*n* = 602, 17%) was labeled the High-relationship quality profile (see Table 2).

The third profile showed mean scores that fell between the Low- and High-relationship quality profiles across most relationship dimensions, with relatively lower intimacy and attachment levels than the High group. This category (*n* = 2,156, 60%) was defined as the Moderate-relationship quality profile (see Table 2).

Descriptive analyses further contextualized these profiles (see Table 3). By gender, the Moderate profile included 56.35% of males and 63.36% of females. In the High profile, males constituted 66.8% and females 33.2%. In the Low profile, males accounted for 22.48% and females 24.97%. By household registration, the Moderate profile comprised 58.65% of urban adolescents and 60.75% of rural adolescents. In the High profile, 56.1% were urban and 43.9% rural, while in the Low profile, the proportions were 23.08% (urban) and 24.28% (rural).

**Table 3:** Distribution of demographic characteristics across latent profiles of teacher–student relationship and friendship quality.

Variable	Low relationship quality profile (*n* = 855)	High relationship quality profile (*n* = 602)	Moderate relationship quality profile (*n* = 2,156)	Test statistic	*P*
Male (%)	22.48%	21.17%	56.35%	*χ*^2^ = 58.58	<0.01**
Female (%)	24.97%	11.67%	63.36%		
Urban (%)	23.08%	18.27%	58.65%	*χ*^2^ = 7.10	<0.05*
Rural (%)	24.28%	14.97%	60.75%		
Grade 7 (%)	25.29%	15.30%	59.41%	*χ*^2^ = 11.64	0.07
Grade 8 (%)	20.72%	15.95%	63.33%		
Grade 10 (%)	24.51%	18.23%	57.26%		
Grade 11 (%)	24.70%	17.34%	57.96%		

M, means; SD, standard deviation.

* *p* < 0.05.

** *p* < 0.01.

Post hoc Scheffé tests clarified the pairwise differences (see Table 2). All three profiles differed significantly for intimacy, attachment, companionship, intimate disclosure, and help & guidance (High > Moderate > Low). For conflict, avoidance, conflict resolution, affirmation & care, and conflict & betrayal, the Moderate profile scored significantly higher than the High profile, and both were significantly higher than the Low profile (Moderate > High > Low).

These findings confirm that relationship quality profiles can be meaningfully distinguished based on mean levels across specific dimensions and demographic distributions such as gender and household registration.

### 3.5 Multinomial logistic regression analysis of latent profiles of teacher–student relationship and friendship quality

Multinomial logistic regression is conducted with the three latent profiles of Teacher–Student Relationship and Friendship Quality as the dependent variable. Gender (female as the reference), household registration (rural as the reference), and interest in physical education are included as predictors (see Table 4). The moderate relationship quality profile is used as the reference category.

**Table 4:** Multinomial logistic regression results predicting profile membership.

Variable	Low relationship quality profile vs. moderate relationship quality profile			High relationship quality profile vs. moderate relationship quality profile		
	B (SE)	OR	95% CI	B (SE)	OR	95% CI
Gender	0.16 (0.11)	1.17	[0.95, 1.44]	0.66 (0.10)	1.93	[1.59, 2.35]
Household Registration	0.00 (0.11)	1.00	[0.81, 1.23]	0.16 (0.10)	1.17	[0.97, 1.42]
interest in physical education	−4.53 (0.18)	0.01	[0.01, 0.02]	1.46 (0.11)	4.29	[3.44, 5.35]

OR, odds ratio; CI, confidence interval.

First, the likelihood of membership in the low relationship quality profile is compared to membership in the moderate relationship quality profile. Table 3 shows a significant negative effect for interest in physical education (*p* < 0.001; OR = 0.01). Specifically, adolescents with lower levels of interest in physical education are much more likely to be in the low relationship quality profile rather than the moderate relationship quality group.

Next, the likelihood of membership in the high relationship quality profile is compared to the moderate group. Significant positive effects are observed for gender (male) (*p* < 0.001; OR = 1.93) and interest in physical education (*p* < 0.001; OR = 4.29). This suggests that male adolescents and those with higher interest in physical education are more likely to be classified into the high relationship quality profile compared to the moderate group.

## 4 Discussion

This study adopted a person-centered approach to examine adolescents' perceived teacher–student and friendship relationships, identifying three distinct profiles of relational quality—low, moderate, and high—and linking these to students' interest in physical education. The findings highlight that relational experiences in schools are heterogeneous rather than uniform, with clear implications for adolescents' engagement in learning and physical activity contexts.

### 4.1 LPA of teacher–student relationship and friendship quality among adolescents

Identifying three profiles—Low-, Moderate-, and High-relationship quality—extends previous research showing that adolescents' perceptions of their social environment are not evenly distributed but cluster in meaningful ways. For example, Wang and Peck (66) identified heterogeneous profiles of school engagement using LPA, demonstrating that subgroups of students follow markedly different trajectories of adjustment. Likewise, Spilt reported that different patterns of teacher–student and peer relationships are linked to adolescents' academic and social outcomes (67). By extending these person-centered approaches to the joint consideration of teacher–student and friendship relationships, the present findings underscore the complexity of students' relational experiences and suggest that multidimensional profiles better capture this heterogeneity than variable-centered methods.

Crucially, this study linked these relational profiles with adolescents' interest in physical education, rather than broader constructs such as general academic motivation. Interest represents a more domain-specific and affective–cognitive orientation, encompassing enjoyment and value attribution toward physical education classes. Previous research has emphasized the role of supportive relationships in enhancing motivation in physical education (19, 22, 68–70), but relatively few studies have examined students' specific interest in physical education as an outcome. Our findings address this gap: adolescents in the High-relationship quality profile reported the strongest physical education interest, whereas those in the Low profile showed substantially lower interest. This suggests that high-quality teacher–student and peer relationships can nurture students' interest in physical education, while relational difficulties are associated with diminished interest.

The results also provide theoretical and practical insights. Theoretically, they highlight the utility of a person-centered lens to capture different constellations of relational experiences and their consequences for physical education interest. This enriches current knowledge by showing that interest—a key yet distinct motivational construct—varies systematically across relational profiles. Practically, the profiles suggest pathways for targeted interventions. Students in the Low-relationship quality profile, who struggle with conflict or limited support, may need relational interventions in physical education classes to build trust and cooperative engagement. For most Moderate profiles, strengthening teacher–student connections and peer support could further enhance their enjoyment and sustained interest in physical education.

In conclusion, this study integrates insights from prior person-centered research on school relationships (67, 71) with the physical education literature (2, 19, 72–74), providing new evidence that adolescents' interest in physical education is closely tied to the quality of their relational experiences. These findings highlight that fostering high-quality relationships in schools is not only vital for socioemotional development but also a key condition for cultivating adolescents' lasting interest in physical education.

### 4.2 Impact of demographic variables on latent profiles of teacher–student relationship and friendship quality among adolescents

This study identified notable gender-related variations in adolescents' perceived relationship quality. Compared with females, males were more likely to be classified into the high-relationship quality profile. Prior research suggests that girls tend to emphasize intimacy, emotional support, and dyadic closeness, whereas boys are more inclined to highlight companionship through shared activities and group participation (75–77). Because activity-based interactions are often positively associated with relationship satisfaction, such orientations may partly explain why male adolescents in this study evaluated their peer relationships as higher in quality.

Beyond these activity-based explanations, self-efficacy may constitute an additional factor. Evidence from physical activity research indicates that males often report higher levels of self-efficacy, which strengthens the link between self-perceptions and engagement behaviors (78). By extension, greater social self-efficacy may enable adolescents to initiate and sustain peer interactions more confidently, thereby perceiving their relationships as higher in quality. Sociocultural factors may also contribute: norms encouraging boys to project confidence and independence may reinforce self-perceptions of strong relational ties (79), whereas girls' heightened emotional sensitivity and reflective awareness may lead them to evaluate relationships more critically, reporting lower quality even when supportive ties are present (80). Taken together, gender differences appear to reflect both developmental tendencies and sociocultural expectations rather than fixed or essentialized traits.

In contrast, no significant urban–rural differences were observed in profile membership. At first glance, this might be surprising given that prior studies sometimes report disparities in adolescents' social resources across urban and rural settings (81, 82). However, recent changes in educational policy and digital connectivity may reduce such differences in relational experiences, making adolescents' perceptions of teacher–student and peer relationships more similar regardless of residence (83). This null finding is consistent with research suggesting that school-based environments increasingly provide comparable relational opportunities across contexts. Key variations within urban and rural communities—such as school quality or class size—may also be more influential than residence itself (84, 85). These explanations imply that while residence has long been considered a salient contextual factor, its role in shaping adolescents' relational profiles may weaken (86). For research on physical education interest, this signals the importance of focusing on proximal relational processes rather than broad demographic categories when explaining variation in students' school experiences.

Beyond demographic differences, it is also vital to interpret the present findings with conceptual precision. Specifically, this study examined interest in physical education, distinct from broader motivational constructs. Interest reflects a relatively enduring, domain-specific orientation involving enjoyment, curiosity, and valuing. In contrast, intrinsic motivation in Self-Determination Theory (11) refers to the situational drive to engage in activities for inherent satisfaction. The two are closely related but not interchangeable: interest can provide the foundation for sustaining intrinsic motivation, yet it remains a distinct construct. Viewed this way, the present results show that adolescents in the high-relationship quality profile reported stronger interest in physical education, not merely greater “motivation”. This clarification enhances the theoretical contribution of our study by positioning interest as a meaningful and independent outcome within the broader literature on adolescent social relationships.

### 4.3 Impact of latent profiles of teacher–student relationship and friendship quality among adolescents on interest in physical education

The present study identified three distinct relational profiles among adolescents—low, moderate, and high relationship quality—systematically linked to their interest in physical education. Adolescents in the high-quality profile reported the strongest interest in physical education, those in the low-quality profile the weakest, with the moderate group situated between these extremes. This pattern underscores the role of perceived relational support as a critical factor in shaping students' willingness to engage with physical education (39, 70, 87–89).

These findings can be interpreted in light of Self-Determination Theory, which highlights the satisfaction of the need for relatedness as a foundation for intrinsic motivation (29, 90–92). When adolescents perceive strong support from teachers and peers, they are more likely to feel connected and to sustain their interest in physical education (39, 57, 70, 88, 93, 94). Conversely, weaker relational perceptions may foster a sense of disconnection that undermines students' interest and engagement (95, 96).

Beyond the general benefits of supportive relational climates, prior research indicates that teacher–student relationship quality plays a decisive role in shaping adolescents' interest in physical education (23, 29, 32, 74). Teachers who adopt autonomy-supportive practices—such as offering meaningful choices, acknowledging students' perspectives, and minimizing controlling behaviors—can strengthen students' sense of relatedness and foster both situational and longer-term physical education interest (97–99). Likewise, peer relationships are critical: acceptance, collaboration, and encouragement from classmates enhance enjoyment and sustained participation in physical education (52, 88, 93, 100), whereas negative dynamics such as exclusion or bullying may erode students' willingness to participate and diminish their physical education interest (101). These insights suggest that schools can support adolescents' physical education interest by cultivating autonomy-supportive instructional practices and intentionally structuring peer interactions to be more collaborative and inclusive.

It is also necessary to acknowledge influences beyond the school context. Studies indicate that adolescents' relational quality and subsequent interest in physical activity are shaped by family climates, including parental support, modeling, and encouragement of autonomy (102–104). Families that value physical activity and provide positive reinforcement may amplify the impact of supportive teacher–student and peer relationships. In contrast, limited family support could constrain the development of strong physical education interest regardless of school environments (105).

Notably, a considerable proportion of adolescents had a moderate profile. Although these students did not perceive very poor relationships, their intermediate levels of support may leave them vulnerable to declines in physical education interest when facing academic or interpersonal stressors. School-based initiatives that strengthen teacher–student bonds and peer collaboration—potentially reinforced by family involvement in adolescents' physical activity—may be especially effective in cultivating and sustaining physical education interest for this group.

By adopting a person-centered approach, this study contributes to understanding how variations in relational quality correspond to adolescents' different levels of interest in physical education. The findings highlight that high-quality relational contexts function as protective resources and as essential conditions for fostering adolescents' sustained interest and enjoyment in physical education, offering practical guidance for educators, schools, and families.

## 5 Limitations

This study has several limitations. First, the cross-sectional design prevents us from drawing causal inferences. For example, it remains unclear whether supportive teacher–student or peer relationships enhance adolescents' interest in physical education, or whether students who are already more interested in physical education are more likely to perceive their relationships positively. Second, all measures were self-reported, which may introduce social desirability bias and recall errors despite the safeguards implemented to encourage honesty and anonymity. Third, convenience sampling from a limited number of schools in one region constrains the generalizability of the findings to broader populations. Moreover, only students from grades 7, 8, 10, and 11 were included. Grades 9 and 12 were excluded due to the unique academic pressures associated with entrance examinations at these levels, which raised logistical challenges for recruitment and follow-up.

Several directions for future research can help address these limitations. Longitudinal designs are needed to establish causal processes linking relational quality to physical education interest more convincingly. In addition, although this study explored gender and urban–rural differences descriptively, we did not formally conduct measurement invariance testing. Future studies should assess whether the identified relational profiles are psychometrically equivalent across demographic subgroups to enable meaningful comparisons. Importantly, multilevel modeling approaches are also recommended, as students are nested within classrooms and schools that systematically shape relationships and opportunities to engage in physical education. Accounting for this clustering will allow researchers to disentangle individual-level effects from contextual-level ones on adolescents' physical education interest. Extending the sample to include grades 9 and 12 would further capture potential turning points in students' interest that coincide with high-stakes examination years.

Research can move beyond descriptive profiling by incorporating longitudinal, invariance, and multilevel approaches in future work to provide more rigorous and context-sensitive evidence. Such advances will clarify how supportive relationships function across developmental and institutional settings and deepen understanding of how these relationships specifically promote adolescents' interest in physical education, thereby extending theory and guiding practical interventions in schools.

## 6 Conclusions

Three relationship quality profiles emerged from the LPA: low, moderate, and high relationship quality profiles. These profiles were significantly associated with differences in adolescents' reported interest in physical education, with students in the high relationship quality profile reporting the greatest interest, those in the low relationship quality profile the least, and those in the moderate relationship quality profile in between. Gender differences were also observed, as boys were likelier to belong to the high relationship quality profile, whereas girls were relatively more represented in the moderate relationship quality profile.

These findings indicate meaningful heterogeneity in adolescents' relational experiences and their systematic associations with physical education interest. By adopting a person-centered approach, this study extends prior work by emphasizing interest in physical education—rather than motivation more broadly—as a key outcome linked to relational contexts. As elaborated in the Discussion, cultivating autonomy-supportive teaching and inclusive peer environments represents a promising direction for practice. Future longitudinal and multilevel approaches will be essential to clarify causal pathways and contextual influences.

## Data Availability

The original contributions presented in the study are included in the article/Supplementary Material, further inquiries can be directed to the corresponding author's.
